# Accelerating the Inbreeding of Multi-Parental Recombinant Inbred Lines Generated By Sibling Matings

**DOI:** 10.1534/g3.111.001784

**Published:** 2012-02-01

**Authors:** Catherine E. Welsh, Leonard McMillan

**Affiliations:** Department of Computer Science, University of North Carolina at Chapel Hill, Chapel Hill, North Carolina 27599

**Keywords:** marker-assisted breeding, mouse, simulation, recombinant inbred lines, Mouse Genetic Resource

## Abstract

Inbred model organisms are powerful tools for genetic studies because they provide reproducible genomes for use in mapping and genetic manipulation. Generating inbred lines via sibling matings, however, is a costly undertaking that requires many successive generations of breeding, during which time many lines fail. We evaluated several approaches for accelerating inbreeding, including the systematic use of back-crosses and marker-assisted breeder selection, which we contrasted with randomized sib-matings. Using simulations, we explored several alternative breeder-selection methods and monitored the gain and loss of genetic diversity, measured by the number of recombination-induced founder intervals, as a function of generation. For each approach we simulated 100,000 independent lines to estimate distributions of generations to achieve full-fixation as well as to achieve a mean heterozygosity level equal to 20 generations of randomized sib-mating. Our analyses suggest that the number of generations to fully inbred status can be substantially reduced with minimal impact on genetic diversity through combinations of parental backcrossing and marker-assisted inbreeding. Although simulations do not consider all confounding factors underlying the inbreeding process, such as a loss of fecundity, our models suggest many viable alternatives for accelerating the inbreeding process.

Recombinant inbred lines (RILs), first developed in 1971 (Taylor *et al.* 1971; Bailey 1971), have long been an important resource for genetics. Typically, RILs are derived by crossing two inbred strains followed by repeated generations of selfing or sibling mating to produce an inbred line whose genome is a mosaic of its parental lines. More recently, panels of multiway RILs have been developed that combine the genomes of multiple founder lines via an initial mixing stage followed by successive generations of inbreeding. Examples include mouse (Threadgill *et al.* 2002; Churchill *et al.* 2004; Chesler *et al.* 2008; Collaborative Cross Consortium 2012), maize (Buckler *et al.* 2009), *Drosophila melanogaster* (Gibson and Mackay 2002), and *Arabidopsis thaliana* (Paulo *et al.* 2008; Kover *et al.* 2009; Gan *et al.* 2011). For all species, inbreeding via either selfing or sibling mating is the primary process used for fixing the genetic background. RILs derived by sibling matings from two parental backgrounds require multiple generations to fix their genome as homozygous, and the number of generations depends on the diploid number. In mice, this requires at least 20 generations (Eisen 2005) and assuming an average of four generations per year, it takes a minimum of 5 years to create a new RIL. Moreover, a large fraction of the started RILs fail, presumably as the result of genetic incompatibilities affecting survival and reproduction (Silver 1995). Many recent efforts to generate RILs have focused on multiway crosses where more than two parental lines are initially mixed before inbreeding. Broman (2005) showed through simulation that eight-way RILs take on average 26.7 generations of sib-mating to reach 99% fixation, and 38.9 generations, on average, to reach complete fixation.

Although a major source of genetic variation in a RIL is derived from the choice of founder strains, we focus on the additional genetic variations introduced by mixing of allele combinations via recombinations between founder genomes. This is the primary source of genetic variation between RILs. Therefore, the number of distinct founder segments, defined as the regions between recombination breakpoints on the RIL chromosomes, can be used as a measure of genetic diversity. From now on, we refer to these distinct founder segments simply as segments. Using simulations, Broman (2005) tracked the number of segments generated through recombination in inbred lines and used it as a comparison between the genetic diversity of two-way and eight-way sib-mating RILs. Recombinations in early generations increase diversity, but eventually diversity peaks and the process of inbreeding leads to a loss of segments. In an eight-way cross, the peak in diversity is reached at the seventh generation of inbreeding on average and before 10 generations of inbreeding for 75% of line starts (Figure 1). Therefore, we will consider 10 generations of inbreeding as past the point of peak diversity. If inbreeding acceleration is started before this peak is reached, the resulting inbred lines are likely to see a reduction in the number of segments. Therefore, unless otherwise specified, we assume a randomized sib-pair mating scheme is used for the first 10 generations, after which we apply various nonmarker-assisted and marker-assisted acceleration techniques.

**Figure 1:**
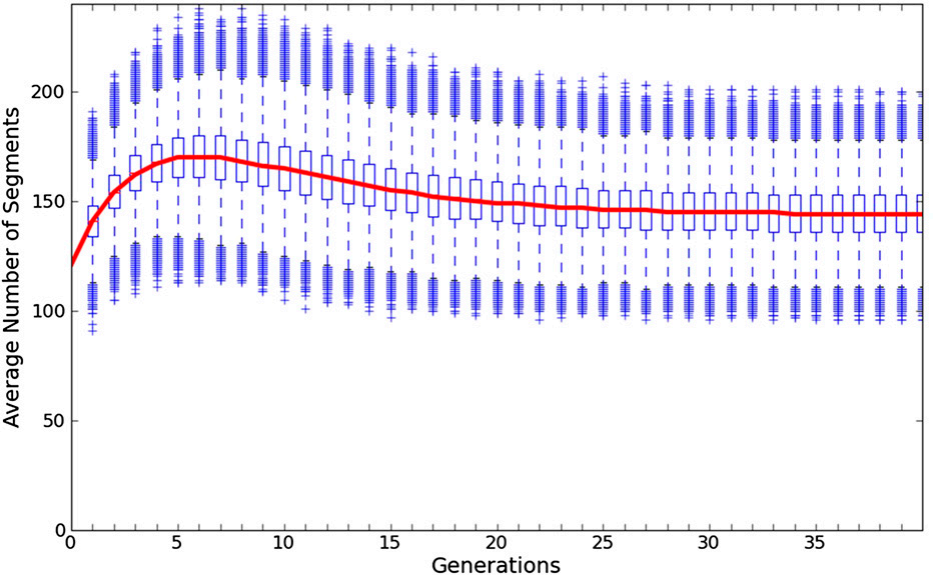
The average number of founder segments in eight-way RILs at various generations of inbreeding. This figure is based on 100,000 simulations, and the number of segments was tracked until they reached complete fixation. The average peak in the number of segments occurs at generation 7 and before generation 10 for 75% of all lines. Therefore, we consider generation 10 to be past the point of peak diversity.

Marker-assisted breeding techniques have been used to fix a selected haplotype interval against a fixed background in congenic strains (Markel *et al.* 1997). In mouse, marker-assisted “speed congenics” have demonstrated a reduction in the number of generations of backcrossing from 10 generations to five. This reduction was achieved by selecting mice that retained the lowest proportion of heterozygous donor to recipient genome. These selection criteria have evolved overtime, as technology has allowed for more rapid genotyping (Flaherty and Bolivar 2007). Just as marker-assisted techniques have been used to improve mapping resolution in self-pollinated species (Boddhireddy *et al.* 2009) and have been adapted for consomics (Armstrong *et al.* 2006), we adapt them for multiparental RILs. Rather than attempt to fix one specific genomic region or one complete chromosome, our goal is to achieve complete fixation of the genome in fewer generations than random sib-matings, without substantially impacting the overall genetic architecture of the inbred lines. In this article, we address accelerating the inbreeding process of outcrossing species by using a combination of alternative breeding strategies and marker-assisted inbreeding (MAI) techniques.

## Materials and Methods

We developed a simulator that represents a genome as a collection of intervals whose boundaries can be resolved at the resolution of a base pair rather than a string of alleles as is common in many breeding simulators (Broman 2005; Valdar *et al.* 2006). The interval representation has the advantage of implicitly representing every base pair in the genome while explicitly tracking every recombination. This approach provides a conservative estimate of homozygosity because it treats every founder sequence as a separate genotype without taking into account regions of sequence identity among founders. Moreover, our interval model can be trivially converted to a string of alleles representation if given the founder sequences or markers from any platform.

**Figure 2:**
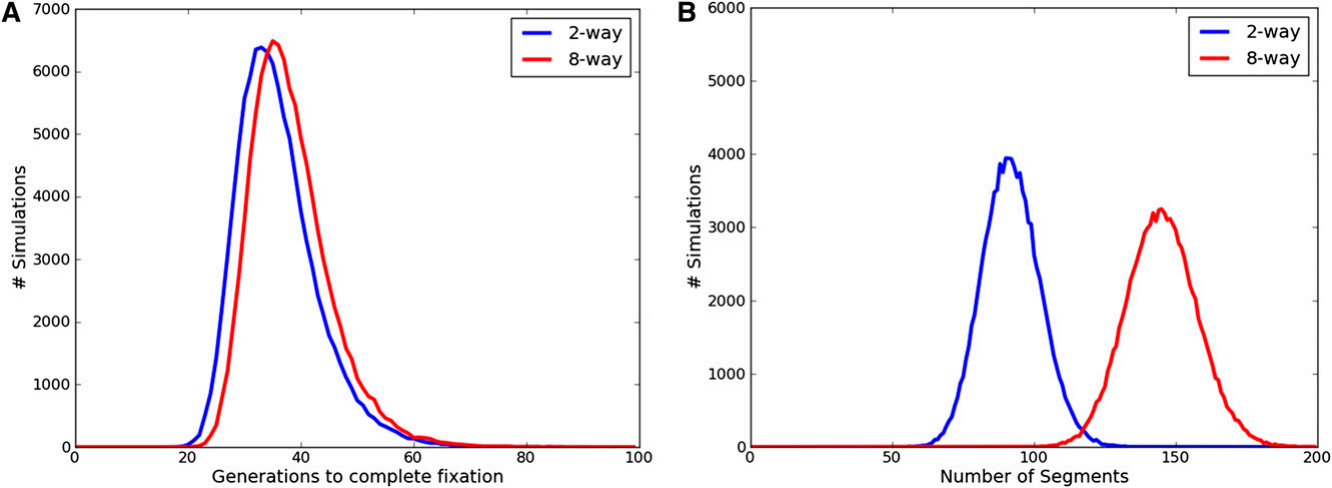
The number of generations to complete fixation (A) and the number of resulting founder segments (B) in two-way and eight-way RILs. On average, two-way RILs take 35.92 generations to reach complete fixation and have 91.95 segments. Eight-way RILs take 38.21 generations and have 145.12 segments on average. These figures are based on 100,000 simulations and are consistent with previous simulations (Broman 2005).

Despite the differences in the underlying representation, our simulator produces results nearly indistinguishable from those presented by Broman (2005). Figure 2 shows the distribution of the number of generations to complete fixation and number of segments for both the two-way and eight-way sib-mating RILs based on the simulation of 100,000 RILs. For a randomized eight-way RIL our simulations show that it takes an average of 38.21 ± 7.1 (SD) generations of sib-matings to reach complete fixation. The genomes of the resultant inbred lines have an average of 145.1 ± 12.48 segments in their mosaic structure. Furthermore, 25.72 ± 3.16 generations of sib-mating on average are needed to reach 99% fixation. These baseline metrics are used for comparison against our accelerated inbreeding simulations.

Our analysis is based on an initial funnel-breeding scheme like that used in the eight-way Collaborative Cross (CC) (Churchill *et al.* 2004; Collaborative Cross Consortium 2012), where the mixing of eight inbred lines occurs in two initial crossing stages (G1 and G2), followed by successive generations of sib-matings (G2:F1, G2:F2, etc) until the line becomes fully inbred. The eight founder strains are represented by letters A-H, and these labels are used to track the descent of genomic segments (see Figure 1) (Collaborative Cross Consortium 2012).

**Figure 3:**
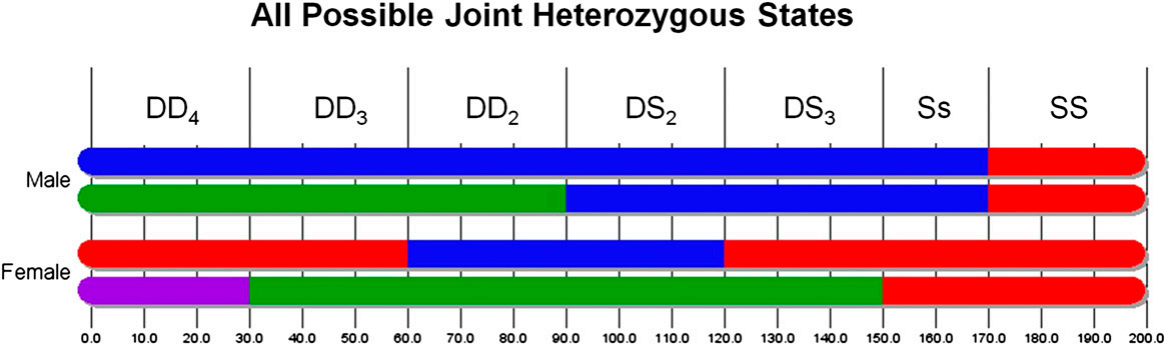
This image shows all possible JH states between a potential mating-pair and illustrates our notion of a genomic segment. DD stands for different-different and occurs in three variations. DD_4_ occurs when both breeders are heterozygous and do not share any founder alleles among them. DD_3_ occurs when both breeders are heterozygous and share one founder allele, whereas DD_2_ refers to both breeders being heterozygous for the same two founder alleles. DS stands for different-same and occurs in two variations. DS_3_ occurs when the heterozygous gene shares no founder alleles with the homozygous allele of its mate. DS_2_ refers to when the heterozygous gene shares one founder allele with its mate. Ss is opposite same, where the male is homozygous for one founder allele and the female is homozygous for another allele. The final state, SS (same-same), is achieved when both male and female are homozygous for the same founder allele. All JH segments are depicted with a chromosome fraction of 0.15, except for Ss, with 0.10.

**Figure 4:**
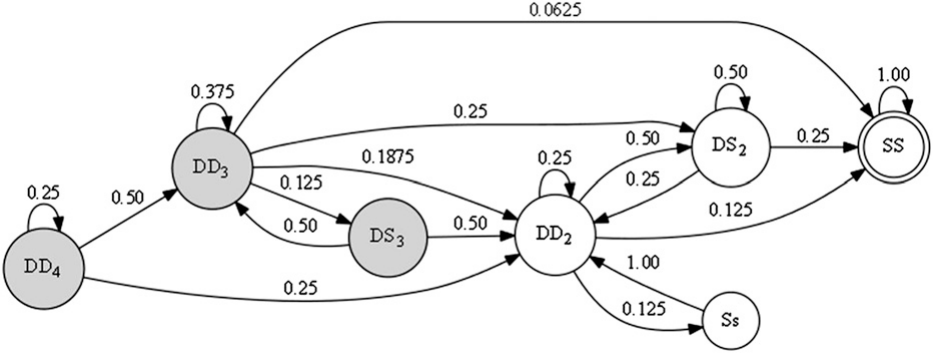
A state diagram showing the transitions between all JH states in a single generation. The directed edges are labeled with the transition probability. The grayed-out nodes represent transient states; once a segment moves away from these three states, there are no returning edges. Transient states tend to go away after a few generations and are rarely seen past the point of peak diversity (as shown in Figure 5). CC lines begin inbreeding in one of the states, DD_4_, DD_3,_, and DD_2_. The desired inbred state for all intervals is SS. DS_2_ is the most likely to become SS. DD_2_ is the next most likely state to become fixed. It takes at least two generations to transit from Ss to SS, as there is no direct path between these two states.

We introduce a notion of joint heterozygosity (JH) to express four possible states between the homologous alleles of a potential breeding pair. Figure 3 shows two homologous chromosomes from each parent of a potential breeding pair and depicts each of the JH states. The inbred state is achieved when both male and female samples are homozygous for the same founder state. We call this state same-same (SS). Another possible state involves a breeding pair that is heterozygous with alleles from two founders while the mate is homozygous. We call this different-same (DS). This state occurs in two forms, DS_2_ when the heterozygous gene shares a founder allele with the homozygous allele of its mate, and DS_3_, when the heterozygous gene shares no founder alleles with its mate. The third state is opposite-same (Ss), where the male is homozygous for one founder and the female is homozygous for another. The final state is different-different (DD), where both male and female are heterozygous. This state comes in three variations, involving, two, three, and four founders, respectively. The two-founder state, called DD_2_, occurs when both male and female are heterozygous between the same founder alleles. DD_3_ refers to when both male and female are heterozygous but share one common founder allele. DD_4_ occurs when the male and female are heterozygous and do not share any founder alleles. Figure 4 shows a state diagram with these four states and their forms depicting all possible transitions between them in a single generation. The directed edge weights represent the probability of transitioning between JH states. A similar transition matrix, which uses 13 states instead of our seven, has also been derived by Broman (2012). It is a simple matter to extend our JH model to two generations by finding every path of length two within the graph and inserting an edge with weight equal to the product of the two edges along its path. The weights of edges from a common source to a common destination, but passing through different intermediate states, can be added and combined into a single edge. This approach can be extended to *n* generations, and as *n* increases all of the heaviest edges eventually lead to the inbred (SS) state. For analytical expressions for extending our JH model for n generations, see (Broman 2012; Collaborative Cross Consortium 2012).

**Figure 5:**
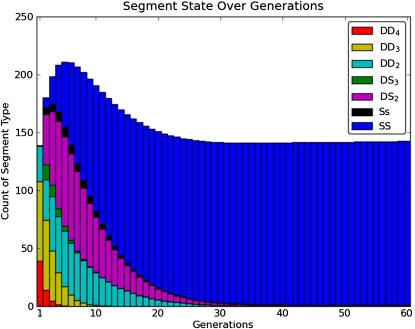
A histogram of segments colored according to their JH state as a function of generation. In early generations, most segments have contributions from three or more founders, but by generation 10 (after the point of peak diversity), segments have contributions from two or fewer founders. This plot was created by tracking the JH states between breeder pairs and finding the average contribution of each state over 100,000 simulations.

In early generations the CC lines include genomic intervals in JH states involving three or more founders (DD_3_, DD_4_, DS_3_), but in later generations these intervals eventually transition to states with two or less founders (DD_2_, DS_2_, SS, and Ss; Figure 5). We can see in Figure 4 that DD_4_, DD_3_, and DS_3_ are transient states, meaning that once this group of three states is left, there are no returning edges. In two-way RILs, the three transient states do not occur because there are at most two founders present. When selfing, the model further reduces to only two JH states, DD_2_ and SS. The transition probabilities to reach the inbred state are incorporated into our metric for selecting the best mating pair at each generation, which is discussed later in this section.

Using the notion of JH state, we split the genome into intervals according to state and track the genomic fraction of each type. We combine these fractions to arrive at several useful measures. Adding the genomic fraction of all regions in the same-same state (SS) gives the fixed genomic fraction (FGF). We call the complement of this, or 1-FGF, the mating pair's combined heterozygous fraction (CHF). FGF and CHF can be used to assess how inbred a line is, such that FGF = 1 refers to fully inbred.

In simulation, we tested a number of modified breeding schemes in an attempt to accelerate the inbreeding process. These nonmarker-assisted breeding schemes minimally impact the traditional RIL generation process. We considered several variations of backcrosses. The use of backcrosses was motivated by the analysis of Broman (2005), which identified a substantial advantage for selfing when compared to sib-mating. Selfing in two-way plant RILs takes on average 10.5 generations to reach complete fixation, which is a substantial reduction from the 35 generations needed when two-way sib-mating.

The steadily decreasing cost of high-density genotyping combined with the advantages of considering each sample's individual full genetic makeup motivated us to also explore MAI techniques. The ability to compare potential breeding pairs based on their high density genotypes allows us to choose breeding pairs with the greatest likelihood of producing inbred offspring. The Ss (opposite same) is the least-preferred state in a breeding pair because it has no chance of becoming inbred in the next generation, as shown in Figure 4. In contrast, of the noninbred states, DS_2_ has the greatest probably of becoming fixed in the next generation, and DD_2_ is the next most likely.

We choose the “best” breeding pair, by considering a weighted genomic mix of the JH types of all candidate mating pairs. The best pair is selected as the maximum of a weighted combination of transition probabilities for all JH segments of a given mating pair considering all chromosomes. For each distinct JH segment of a chromosome the probability that it will become inbred in the next generation (*i.e.*, the weight of the edge from the current JH state to the SS state) is multiplied by the chromosome fraction of the segment, and the sum is accumulated over all segments on the chromosome. This calculation results in a chromosome score ranging from 0, when the entire chromosome is Ss, DD_3_, DD_4_, or DS_3_, to 1 when the entire chromosome is SS. This approximation ignores the relative ordering of segments, and, therefore, does not consider linkage. The individual chromosome scores are then multiplied together, modeling their independent segregation, to arrive at the total pair score. Therefore, we assign a score for a given mating pair as:

$$\mathrm{Score}\left(n,m\right)=\prod_{i=1}^{N}\sum_{JHSeg_{n,m}\in Chr_{i}}p({\mathrm{JHSeg}}_{n,m}\to SS)\frac{\left\|JHSeg_{n,m}\right\|}{\left\|Chr_{i}\right\|}$$

This score is an approximation of the actual likelihood that the entire genome will become inbred in the next generation. We refer to this score as the weighted state metric (WSM). *JHSeg_n,m_* represents a JH segment on the specified chromosome *i* induced by the pairing *n,m*, and the best pair is the maximum of this score over all possible pairs *n,m*. In self-pollinated species, our score simplifies to a scaled version of the FGF because the only relevant states are DD_2_ and SS, which has been described previously (Boddhireddy *et al.* 2009).

We explored two marker-assisted breeding schemes. The first of these is MAI, which modifies the breeding scheme only after the point of peak diversity is reached. Once the peak is reached, the WSM discussed previously is applied to choose the best breeding pairs. The second is a marker-assisted advanced intercross, which modifies the breeding scheme to choose sib-pairs to increase segments until either a specified generation or a desired number of segments is reached; it then reverts to choosing sib-pairs to accelerate inbreeding. Through simulations, we track the average number of generations to fully inbred and to 99% inbred as well as the average number of segments present in the inbred lines to compare the different breeding schemes.

The simulator is written in Python and runs on a Dell Studio XPS with 8GB RAM, with dual-threaded quad-core processors. It takes approximately 5.5 hr to complete 100,000 simulations of eight-way RILs.

For the purposes of this analysis, the eight-way CC funnel breeding scheme was used, but our simulator also supports the input of any breeding scheme using pedigree files. It has also been used to simulate two-way RILs, F2 crosses, and outbred populations.

To test our MAI methods, we used the developing CC (Collaborative Cross Consortium 2012) and a low-density genotyping platform we designed, referred to as the Mouse Universal Genotyping Array (MUGA). The SNPs on MUGA are evenly distributed with an average spacing of 325 Kb and a standard deviation of 191 Kb. In an eight-way cross, the genotypes at multiple markers (at a minimum three) are needed to distinguish among the founders. The founder assignments and recombination breakpoints are inferred from the genotypes using a hidden Markov model similar to the ones described by Mott *et al.* (2000), Zhang *et al.* (2009), and Liu *et al.* (2010). Because multiple markers are needed to distinguish each founder, the effective founder-ascertainment resolution of MUGA is approximately 1 Mb.

## Results

### Nonmarker-assisted breeding schemes

**Figure 6:**
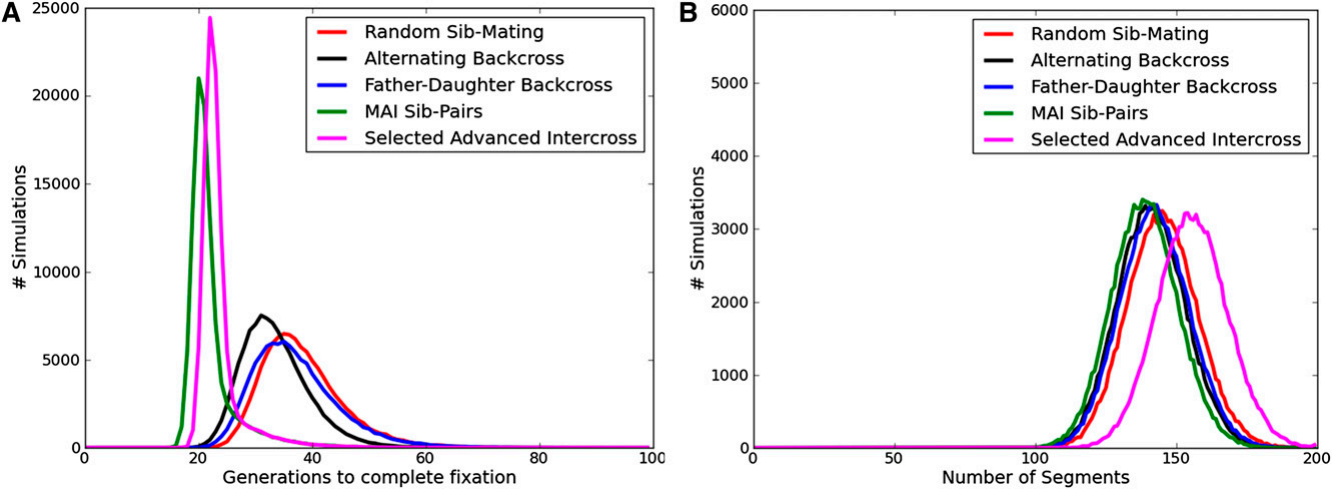
A comparison of five breeder selection alternatives for generating an eight-way RIL, showing the number of generations to reach complete fixation (A) and the total number of segments (B) found in the final inbred lines. Random sib-pair mating is used as our baseline. The alternating backcross swaps between father−daughter and mother−son matings in successive generations. The father−daughter scheme alternates between father−daughter and random sibling matings in successive generations. MAI uses our weighted state metric to choose between 16 breeding pairs after the point of peak diversity. The selected advanced intercross modifies early stages of the breeding scheme to choose sib-pairs that maximize diversity, and then at a pre-established generation (10), it reverted to choosing sib-pairs to accelerate the inbreeding process.

The first breeding scheme examined was alternating back-crosses in successive generations, father-to-daughter in one generation followed by mother-to-son in the next (supporting information, Figure S1). This scheme has many practical advantages in that it leverages known-fertile samples. Furthermore, this strategy also serves as a useful fallback for preserving lines. We simulated this approach starting after the point of peak diversity, with a backcross between a father and daughter followed by a backcross between a mother and son in the next generation (each breeder is used in two successive generations, alternating dam and sire). This process was repeated for each subsequent generation until complete fixation was achieved. Alternating backcrosses achieves a reduction in the number of generations to complete fixation with an average number of generations of 33.45 ± 5.88 (Figure 6). This represents a reduction of nearly five generations over randomized mating and a substantial reduction in variance. It decreases the number of segments in the resulting inbred lines to 141.21, a loss of about four segments on average. The alternating backcross also reduces the number of generations to 99% fixation to 23.45 ± 3.11, a reduction of two generations.

There are several practical limitations to the alternating backcrossing approach. For instance, female fertility often spans a limited window that might not allow for mother-son backcrossing. Therefore, we also explored, through simulation, a modified breeding scheme involving only father-daughter backcrosses. Starting after the point of peak diversity, a father-daughter backcross is followed in the next generation by a random sib-mating. This breeding scheme (Figure S1) is repeated for each subsequent generation until complete fixation is achieved. The father-daughter backcross takes 37.06 ± 7.55 generations to reach complete fixation, and the inbred lines contain on average 142.39 ± 12.24 segments (Figure 6). This breeding scheme also takes 24.70 ± 3.54 generations to 99% fixation. Although the benefits of father-daughter mating appear modest relative to random sib-mating, in practice they are compensated for by a reduction in generation time resulting from a mature and known fertile sire.

### Marker-assisted inbreeding

For all MAI techniques, random sib-matings were simulated until the point of peak diversity was passed. This was followed by subsequent generations of selecting the best breeding pair, until the line reached complete fixation.

Using the WSM, we selected the best pair from sib-pairs, parent−child backcrosses, or a combination of both. To see what other pair relationships were worth considering, we simulated 100,000 lines such that random sib-matings were used for 15 generations, at which time three mating pairs were generated, producing two male and two female offspring each. The best breeding pair was then chosen by comparing every female to every male (both parents and offspring). The pair with the lowest CHF was selected. Sib-pairs were selected 63% of the time, whereas backcrosses were chosen 23% of the time. Cousin-pairs (offspring from different mating pairs of the same generation) were the next most likely, being selected 6.9%. The remaining 7.1% included mating combinations such as aunt−nephew, uncle−niece, or grandparent−grandchild. We concluded that non-sib, non-backcross matings should be used sparingly, except in the case of preserving a line.

Because sib-pairs were most often the best option, we limited subsequent simulations to selecting the best sib-pair and report those statistics in Figure 6 and Table 1. For the MAI sib-pairs breeding scheme, random sib-matings were simulated until the point of peak diversity was reached. After this point, four female and four male offspring were simulated (4-4), all pairs were considered, and the best pair was chosen as the breeders. This process was continued until the line reached complete fixation. Our model is based on generation number and may require multiple litters to achieve the four females and four males assumed in simulation. A potential shortcoming of our model is that we report the time to inbred as a function of generations, not the number of litters or calendar time required to produce enough viable offspring. However, we did perform additional simulations assuming smaller litter sizes (two females, two males), and unbalanced sex-ratio (eight total offspring with one to seven females), and compared all three sets of assumptions (4-4, 2-2, 8) to the greedy approach of setting up breeders as soon as any sibling mating pairs are available. Any form of MAI was always able to considerably reduce the number of generations to achieve inbred status regardless of sex balance or litter size. Moreover, waiting for a sufficiently large breeder-candidate set always outperformed the greedy approach of setting up matings as soon as any pair was available. More details on this analysis appear in the supplementary documentation (Figure S2).

**Table 1:** Number of generations to 100% fixation (fully inbred), 99% fixation, and number of segments for different breeding schemes

Breeding Scheme	Average Generations to Fixation	SD Gens to Fixation	Average Generations to 99% Fixation	SD Gens to 99% Fixation	Average No. Segments	SD, No. Segments
Two-way	35.92	7.13	23.47	3.19	91.95	10.21
Eight-way random sib-pairs	38.21	7.10	25.72	3.16	145.12	12.48
Alternating backcross	33.45	5.88	23.45	3.11	141.21	12.07
Father−daughter backcross	37.06	7.55	24.70	3.54	142.39	12.24
MAI	22.10	4.41	16.44	1.00	138.83	11.83
Marker-selected advanced intercross	23.54	3.82	18.45	0.88	155.63	12.53

SD, standard deviation; MAI, marker-assisted inbreeding.

**Figure 7:**
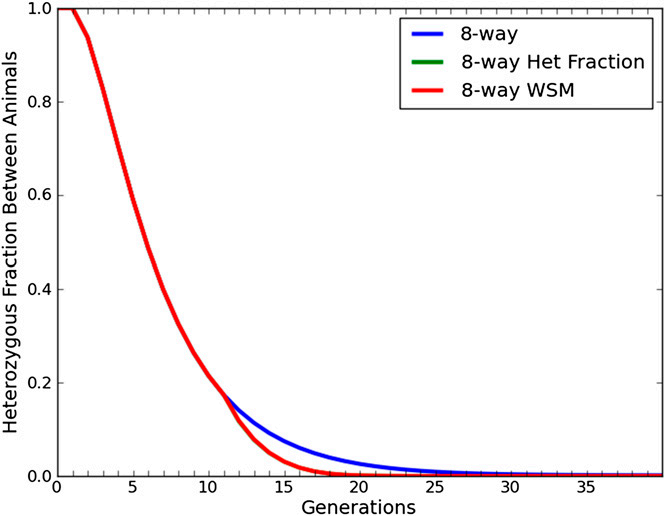
CHF as a function of number of generations. This plot shows that MAI reduces the CHF among breeding pairs much faster than random sib-matings. We can see the effect as soon as the breeding scheme is modified (at the point of peak diversity).

Using this MAI breeding scheme, it was found that 99% fixation can be reached in an average of 16.44 ± 1.00 generations, whereas complete fixation can be reached in 22.10 ± 4.41 generations on average. These inbred lines have an average of 138.83 ± 11.83 segments. Figure 7 shows that MAI reduces the CHF among mating pairs much faster than random sib-matings. As soon as the breeding scheme is altered at the point of peak diversity, the effect is apparent.

### Selected advanced intercrosses

Although MAI achieves a substantial reduction in the number of generations required to fix an RIL, it does so with an average loss of approximately seven segments per line. This result is unfortunate because the number of segments determines the resolution of a RIL panel for quantitative trait mapping (Aylor *et al.* 2011). Therefore, we attempted to overcome this loss by using marker-assisted techniques in the first 10 generations of inbreeding to select mating pairs most apt to increase the number of recombination segments. We refer to these lines as selected advanced intercrosses (Darvasi and Soller 1995) in that they attempt to increase the number of segments on every chromosome by maximizing diversity until a designated generation is reached. This is similar to work done in self-pollinating populations to maximize mapping resolution (Boddhireddy *et al.* 2009). After the designated generation, the same MAI techniques as discussed previously are used to select the breeding-pairs until the line is fixed. We found that it took on average 23.5 ± 3.82 generations to become inbred. At the point of peak diversity, the lines had an average of 196.1 ± 15.44 segments, compared with 167 segments in randomized sib-pair matings. The average number of segments in the final inbred animals was 155.6 ± 12.53. On the basis of our analysis, if genotyping is done at every generation, the lines will become inbred in approximately the same number of generations as the MAI breeding strategy but will have approximately 17 more segments per animal. This could lead to increased mapping resolution in the final population.

### Low-resolution sampling

In our MAI analysis, we assumed that one is able to accurately assign genomic regions to founders at single base-pair resolution. In reality, genotyping platforms have a limited resolution with which they can ascertain a founder's genomic sequence. This limited resolution creates two main obstacles to the use of MAI methods: the possibility that small recombination intervals might escape detection, and the imprecision with which the cross-over points of recombination can be detected. The impact of both of these limitations can, however, be modeled in a simulation.

We modeled this reduced resolution by sampling the JH state at 1-Mb intervals. We simulated the breeding using the MAI breeding strategy discussed earlier, but modified the WSM to consider the JH state only at sample points. Furthermore, we declared lines inbred on the basis of the 1-Mb sampling (when all sample points were SS). We then inspected each declared “inbred” mouse to see if, at a base-pair resolution, all intervals were truly fixed, and found them to be actually inbred only 38.3% of the time. On average we missed three nonfixed segments per line, and these segments were on average 327± 234 Kb. We also found that the lines were considered inbred approximately 2.5 generations earlier than MAI with complete observability. This finding implies that the inability to detect small recombinants might require additional inbreeding generations to attain the desired level of fixation.

## Discussion

Through simulations, we have developed several alternatives to random sib-matings to dramatically accelerate the creation of RILs by as much as 16 generations. These include the judicious use of parental backcrossing and the selection of mating pairs based on genotypes from genome-wide SNPs. Both of these techniques, when applied after the point of peak diversity is reached, result in a negligible reduction in the number of segments. We also propose an advanced intercross variant in which MAI is applied during the early generations to increase the number of haplotype segments for better mapping resolution.

In simulation we also have the luxury of assuming uniform litter sizes and equal sex ratios, but in reality the fecundity of a RIL and the sex-balance of litters are complicating issues. As lines become more inbred, fertility generally decreases (Silver 1995). One way to address this is to use backcrosses as discussed previously. However fertility issues might override the choice of “best breeding pair.” To address this problem we calculate backups that, when used, may extend the number of generations required to achieve fixation.

Taking fertility into account and prioritizing for the preservation of the lines, how do we select the final breeders? WSM optimizes for becoming inbred in one generation, but it might be more advantageous in the early MAI generations to select for animals whose probability to become inbred in two or more generations is maximized. However, in simulations, the two-generation metric generally chooses the same breeding pairs as the single-generation model, leading to the same number of generations to achieve fixation. Once lines reach small levels of residual heterozygosity, it might also be advantageous to maintain multiple breeding pairs selected to produce compatible offspring, which are more like sib-pairs than cousin-pairs. This provides more pair options, as well as a chance to compensate for uneven sex ratios or small litter sizes. Although it seems best to choose the optimal breeding pairs early on, finding good pairs near the end-game (fixing the last 1%–2% of the genome) is a harder problem. The last few heterozygous regions can take several generations to fix if compatible breeding pairs do not exist.

The simulation software used in this analysis is available for download from http://sourceforge.net/p/breedingsim/. It has been adapted for many uses other than marker assisted inbreeding such as estimating the significance of measured statistics in the developing CC (Collaborative Cross Consortium 2012).

## Supplementary Material

Supporting Information
