# Supplementary material for: Accelerating the Inbreeding of Multi-Parental Recombinant Inbred Lines Generated By Sibling Matings
Source: G3 (Bethesda). 2012 Feb 1;2(2):191–8. doi: 10.1534/g3.111.001784 (PMC3284326; doi:10.1534/g3.111.001784)
Supplement: Supporting Information [file supp_2_2_191__index.html]

Supporting Information 

# Accelerating the Inbreeding of Multi-Parental Recombinant Inbred Lines Generated By Sibling Matings

## Supporting Information for Welsh and McMillan, 2012

**Files in this Data Supplement:**

- Supporting Information - Figures S1 and S2 (PDF, 246 KB)
- Figure S1 - This figure shows the pedigree diagrams for the alternating backcrosses: father-daughter backcross with the mother-son backcross (A) and the father-daughter with the random sib-mating (B) (PDF, 57 KB)
- Figure S2 - Compares the number of generations it takes to achieve complete fixation for 5 breeding schemes that make different assumptions about the available pool of breeders (PDF, 193 KB)
