# Supplementary material for: Accelerating the Inbreeding of Multi-Parental Recombinant Inbred Lines Generated By Sibling Matings
Source: G3 (Bethesda). 2012 Feb 1;2(2):191–8. doi: 10.1534/g3.111.001784 (PMC3284326; doi:10.1534/g3.111.001784)
Supplement: Supporting Information [file supp_2.2.191_FigureS1.pdf]

BACKCROSSING PEDIGREES

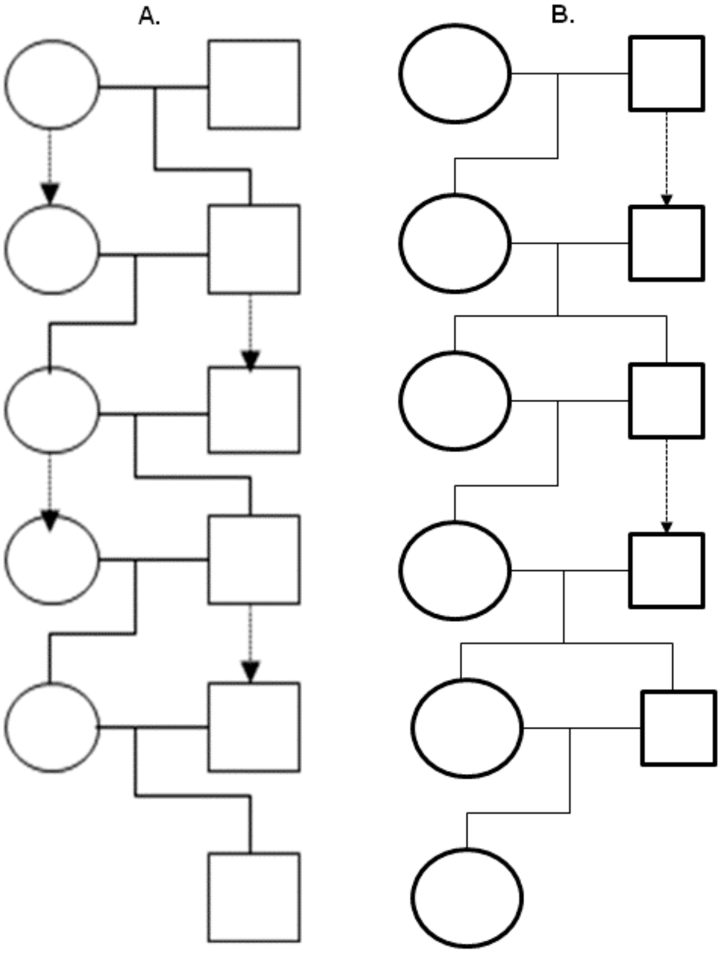

Figure S1 This figure shows the pedigree diagrams for the alternating backcrosses: father-daughter backcross with the mother-son backcross (A) and the father-daughter with the random sib-mating (B).
