# Supplementary material for: Accelerating the Inbreeding of Multi-Parental Recombinant Inbred Lines Generated By Sibling Matings
Source: G3 (Bethesda). 2012 Feb 1;2(2):191–8. doi: 10.1534/g3.111.001784 (PMC3284326; doi:10.1534/g3.111.001784)
Supplement: Supporting Information [file supp_2.2.191_FigureS2.pdf]

ALTERNATE LITTER-SIZE ASSUMPTIONS

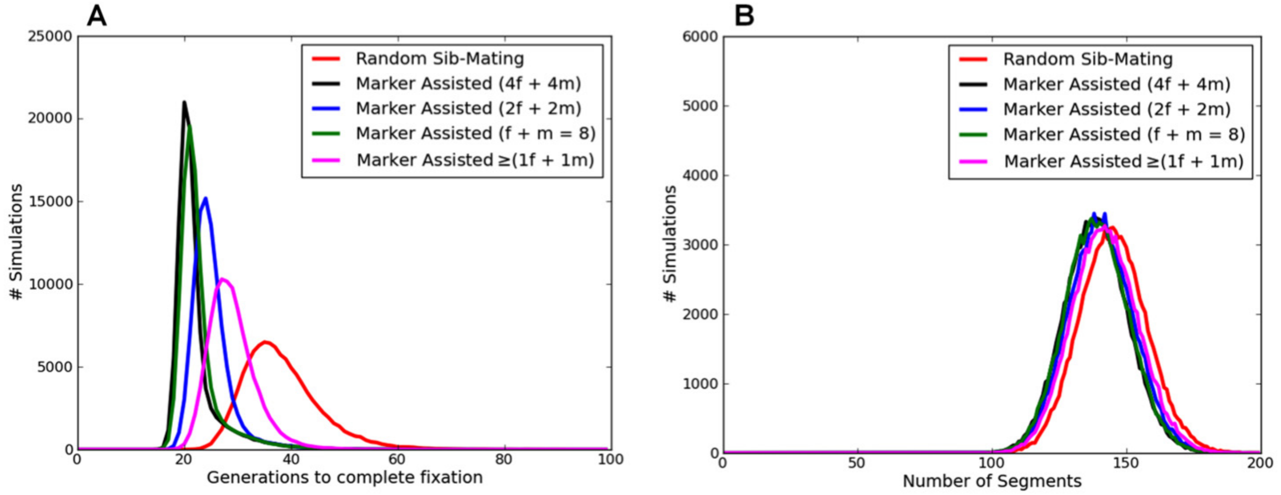

Figure S2 Compares the number of generations it takes to achieve complete fixation for 5 breeding schemes that make different assumptions about the available pool of breeders. The standard random sib-mating is provided for comparison. MAI Sib-Pairs assuming a pool of 8 breeders with 4 of each sex and takes an average of 22.10±4.41 generations to reach complete fixation. The MAI Sib-Pairs assuming a pool of 4 (2 each sex) takes an average of 25.05±3.89 generations to reach complete fixation. The MAI Sib-Pairs Unbalanced Sex-Ratio assumes 8 offspring with varying sex-ratios at each generation. These sex-ratios range from 1 female and 7 males to 7 females and 1 male. This breeding scheme requires an average of 22.63±4.25 generations to reach complete fixation. Finally, the Greedy Sib-Pairs (Marker Assisted ≥ (1f + 1m)) breeding scheme creates small litters of 1-3 offspring and sets up the best breeder pair as soon as at least 1 female and 1 male offspring exist. The Greedy Sib-Pairs breeding selection depicts the natural inclination to set up breeders as soon as possible; however, our simulations indicate that it does not reduce the number of generations required to reach complete fixation as much as waiting until 8 offspring are available for comparison. In fact, it requires an average of 28.97±4.46 generations to reach complete fixation. The overall impact of each breeding scheme on the genetic diversity is negligible.
